# Gene markers for exon capture and phylogenomics in ray‐finned fishes

**DOI:** 10.1002/ece3.5026

**Published:** 2019-03-05

**Authors:** Jiamei Jiang, Hao Yuan, Xin Zheng, Qian Wang, Ting Kuang, Jingyan Li, Junning Liu, Shuli Song, Weicai Wang, Fangyuan Cheng, Hongjie Li, Junman Huang, Chenhong Li

**Affiliations:** ^1^ Shanghai Universities Key Laboratory of Marine Animal Taxonomy and Evolution, Key Laboratory of Exploration and Utilization of Aquatic Genetic Resources (Shanghai Ocean University), Ministry of Education, Shanghai National Demonstration Center for Experimental Fisheries Science Education (Shanghai Ocean University) Shanghai China

**Keywords:** Actinopterygii, bait design, nuclear gene markers, phylogenomics, population genomics, target enrichment

## Abstract

Gene capture coupled with the next‐generation sequencing has become one of the preferred methods of subsampling genomes for phylogenomic studies. Many exon markers have been developed in plants, sharks, frogs, reptiles, fishes, and others, but no universal exon markers have been tested in ray‐finned fishes. Here, we identified a suite of “single‐copy” protein‐coding sequence (CDS) markers through comparing eight fish genomes, and tested them empirically in 83 species (33 families and nine orders or higher clades: Acipenseriformes, Lepisosteiformes, Elopomorpha, Osteoglossomorpha, Clupeiformes, Cypriniformes, Gobiaria, Carangaria, and Eupercaria; sensu Betancur et al. 2013). Sorting the markers according to their completeness and phylogenetic decisiveness in taxa tested resulted in a selection of 4,434 markers, which were proven to be useful in reconstructing phylogenies of the ray‐finned fishes at different taxonomic levels. We also proposed a strategy of refining baits (probes) design a posteriori based on empirical data. The markers that we have developed may greatly enrich the batteries of exon markers for phylogenomic study in ray‐finned fishes.

## 1 INTRODUCTION

Next‐generation sequencing (NGS) drastically reduced the cost of sequencing a genome, so that reconstructing phylogenetic relationships using whole genomes became feasible (Jarvis et al., 2014). However, sequencing whole genomes is still costly and sometimes unnecessary. Subsampling genome sequences has gained popularity in phylogenetics and population genomics in recent years (Emerson et al., 2010; Faircloth et al., 2012; Lemmon, Emme, & Lemmon, 2012; Li, Hofreiter, Straube, Corrigan, & Naylor, 2013; Peterson, Weber, Kay, Fisher, & Hoekstra, 2012). There are mainly two different genome subsampling tools. One is associated with restriction site‐related markers, such as restriction site‐associated DNA (RAD; Baird et al., 2008) and double digest RADseq (ddRAD) markers (Peterson et al., 2012), which could be used to produce sequences from a tremendous number of anonymous loci that are particularly useful in studying population genomics or species‐level phylogeny (Davey & Blaxter, 2010). The other method is gene capture, also known as target enrichment to capture and sequence target loci, which often result in less missing data than the restriction site‐related methods do (Collins & Hrbek, 2015), and the target loci can be applied across highly divergent taxonomic groups (Faircloth et al., 2012; Lemmon et al., 2012; Li et al., 2013). Benefitting from the advantages of two methods, hybrid approaches (Ali et al., 2016; Hoffberg et al., 2016) have also been developed resulting in less missing data and higher coverage compared with traditional RADseq approaches.

Gene capture is based on hybridizing RNA/DNA baits (probes) to DNA libraries of targeted species and enriching sequences similar to the baits for subsequent high‐throughput sequencing. Two popular methods, Ultraconserved Element (UCE) captures (Faircloth et al., 2012) and Anchored Hybrid Enrichment (AHE; Lemmon et al., 2012), were developed to retrieve single‐copy highly conserved elements in the genome along with variable flanking regions. A third method, exon capture was designed explicitly to capture single‐copy coding sequences across moderate to highly divergent species (Bi et al., 2012; Hedtke, Morgan, Cannatella, & Hillis, 2013; Li et al., 2013). Exons have been more commonly used for phylogenetics than anonymous noncoding regions, and evolution of protein‐coding sequences has been well studied. Furthermore, lowered stringency in hybridization and washing steps enables baits to hybridize with more distant sequences, so it solves the problem that divergent baits and targeted exons may produce missing data (Cosart et al., 2011 and Mason, Li, Helgen, & Murphy, 2011).

Exon capture markers have been developed in plants (Chamala et al., 2015; Mandel et al., 2014; Weitemier et al., 2014), invertebrates (Hugall, O'Hara, Hunjan, Nilsen, & Moussalli, 2016; Mayer et al., 2016; Teasdale, Kohler, Murray, O'Hara, & Moussalli, 2016; Yuan et al., 2016), and many vertebrate groups, including sharks and skates (Li et al., 2013), frogs (Hedtke et al., 2013; Portik, Smith, & Bi, 2016), skink lizards (Bragg, Potter, Bi, & Moritz, 2016), and others. As the most diverse group of vertebrates with more than 30,000 described species (Nelson, Grande, & Wilson, 2016), many studies applied target enrichment to investigate the phylogenetic relationships of ray‐finned fishes (Actinopterygii), but most of them focused on using UCE markers (Chakrabarty et al., 2017; Faircloth, Sorenson, Santini, & Alfaro, 2013; Gilbert et al., 2015; Harrington et al., 2016; Hulsey, Zheng, Faircloth, Meyer, & Alfaro, 2017; Longo et al., 2017; McGee et al., 2016). As a complementary approach, many exon markers have been reported previously for some ray‐finned fishes. Ilves and Lopez‐Fernandez (2014) developed 923 exon markers for cichlids based on genome sequence of *Oreochromis niloticus*. Arcila et al. (2017) tested 1,051 exon markers on the Otophysii. We also developed 17,817 single‐copy nuclear coding sequence (CDS) markers and applied those in the sinipercid fish in a previous study (Song, Zhao, & Li, 2017). However, those makers have not been tested on other groups and may not work well across all ray‐finned fishes. Hughes et al. (2018) selected 1,721 exon markers >200 bp from the 17,817 markers and retrieved their sequences from hundreds of transcriptomic and genomic datasets in silico, although they did not verify their utility in wet laboratory experiments.

Selecting target markers and designing baits that are effective across a wide range of species is the first major challenge when applying the gene capture method. Many considerations are taken into baits design, such as uniqueness and conservativeness of markers, length and complexity of markers, and genetic distance between baits and target sequences (Bi et al., 2012; Campana, 2017; Faircloth, 2017; Faircloth et al., 2012; Gilbert et al., 2015; Hugall et al., 2016; Lemmon et al., 2012; Li et al., 2013; Mayer et al., 2016). However, all these measures are usually taken a priori, and few studies have been done to refine baits design after gene capture to improve the baits set for future experiments (but see Branstetter, Longino, Ward, & Faircloth, 2017).

In this study, we tested the 17,817 CDS previously identified as a part of a separate study (Song et al., 2017) and screen these markers to identify the best ones for inferring phylogeny across all major clades of ray‐finned fish. We chose phylogenetically decisive markers based on the results of pilot experiments and refined the bait design to improve evenness of reads coverage across all loci. Finally, we tested phylogenetic usefulness of selected markers in ray‐finned fishes at both order level and species level. Our goal is to provide a set of common exon markers for gene capture and phylogenomic studies in the ray‐finned fishes.

## 2 MATERIALS AND METHODS

### 2.1 Identification of original marker sets and collecting preliminary data for candidate markers

**Figure 1:**
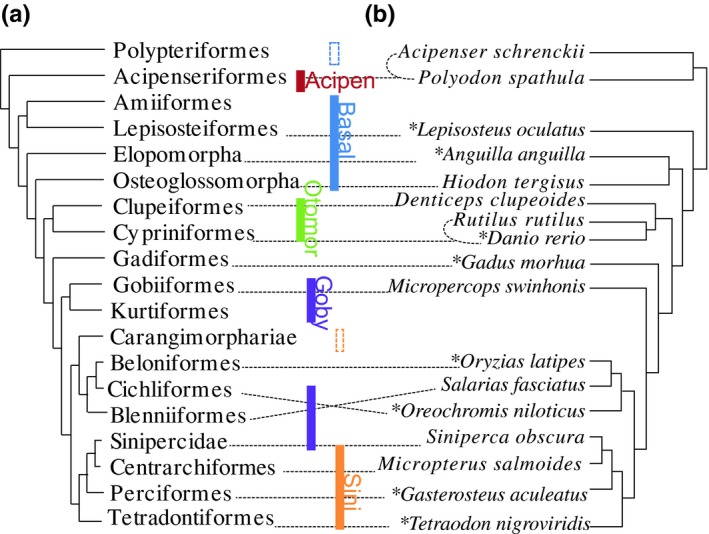
(a) Phylogenetic relationships among 21 groups of ray‐finned fish (Betancur et al., 2013; Hughes et al., 2018). The vertical bars indicate different projects carried in the author's laboratory. The unfilled vertical bars indicate groups that captured <3,000 loci. (b) Maximum likelihood tree of 17 representative ray‐finned fishes based on 4,434 exon loci, all nodes have a 100 bootstrap value. The connected dotted lines between two trees indicate the corresponding taxa. Eight species names marked with stars indicate the fishes used in finding the target markers

The markers were identified through comparing eight fish genomes (Figure 1a) using a bioinformatics tool, EvolMarkers (Li, Riethoven, & Naylor, 2012; Supporting information Appendix S1: Figure S1). Two sets of single‐copy markers were generated and used to design baits to capture species in five different research projects conducted in the authors’ laboratory, including works on early‐branching actinopterygians lineages (Basal), acipenseriforms (Acipen), otomorphs (Otomor), gobioids (Goby), and sinipercids (Sini) (Supporting information Appendix S1: Figure S2). One set of markers was designed based on *Oreochromis niloticus* including 17,817 loci (used in “Goby” and “Sini” projects). The other one was identified from *Lepisosteus oculatus* comprising 13,843 loci (used in “Basal,” “Acipen” and “Otomor” projects).

Thousands of the candidate CDS markers were tested empirically as pilot experiments in 83 actinopterygian species (99 individuals, 33 families of nine orders or higher clades), covering major clades of ray‐finned fishes (Supporting information Table S1).

According to suggestion of the manufacturer, biotinylated RNA baits (MYcroarray, Ann Arbor, Michigan) were synthesized with 2× tiling. Because thymine and adenine have fewer hydrogen bonds with its complimentary nucleotide compared with cytosine and guanine, the 3′ end of the baits was padded with “Ts” if baits were shorter than 120 bp. For the baits designed on *O. niloticus*, loci longer than 100 bp were targeted. For the baits designed on *L. oculatus*, loci longer than 120 bp were targeted.

Total genomic DNA was extracted from fin or muscle tissue of samples using a Tissue DNA kit (Omega Bio‐tek, Norcross, GA, USA) and quantified using a NanoDrop 3300 Fluorospectrometer (Thermo Fisher Scientific, Wilmington, DE, USA). Samples of 350–500 ng genomic DNA were sheared to ~250 bp using a Covaris E220 Focused‐ultrasonicator (Covaris, Woburn, USA). Subsequently, sheared DNA was used to construct libraries. Blunt‐end repair, adapter ligation, fill‐in, prehybridization PCR, and double exon enrichment steps mainly followed the protocol of cross‐species gene capture (Li et al., 2013). The enriched libraries were amplified in 25 μl PCR reactions with a forward primer that included 8 bp custom designed indices, a reverse primer, and KAPA HiFi taq ready mix (Kapa Biosystems, Wilmington, MA, USA). Custom indices with at least two nucleotide differences among the indices were designed following Meyer and Kircher (2010). The concentration of products was measured using a NanoDrop 3300 Fluorospectrometer. The products were pooled in equimolar concentrations and sequenced on an Illumina HiSeq 2500 platform (Illumina, Inc, San Diego, CA, USA) with other samples from the same or other projects at Annoroad (Beijing, China).

Read assembling followed the pipeline of Yuan et al. (2016) except that contigs and respective homologous bait sequences were translated into amino acid sequences before comparison. The raw reads were parsed to respective files for each species according to the 8 bp indices on the P7 adaptor using BclToFastq (Illumina, Inc). The remaining adaptor sequence on the 3 primer end and low quality bases were trimmed from raw reads using Trim_galore v0.4.1 (http://www.bioinformatics.babraham.ac.uk/projects/trim_galore/) with default parameters. Then, PCR duplicates were filtered and parsed to homologous bait sequences. Reads were separately assembled into contigs using Trinity v2.0.6 (Grabherr et al., 2011) with default parameters. Overlapped contigs were further assembled using Geneious v7.1.5 (Kearse et al., 2012). Each contig was translated into amino acid sequences and compared with the amino acid sequences of the original baits using the Smith–Waterman algorithm (Smith & Waterman, 1981). The most similar match to each bait was selected as putative target sequence. The section of the target sequence covering the bait sequence in the alignment was identified as the exon, and the remaining was considered flanking sequence. To identify potential paralogs in retrieved sequences, we used BLAST (Altschul, Gish, Miller, Myers, & Lipman, 1990) to align them against the genomes of *O. niloticus* or *L. oculatus*. Sequences with the best BLAST hit not in target region of the genomes were recognized as potential paralogs and excluded from further analysis. All steps were automated using custom Perl scripts except the further assembly of overlapped contigs in Geneious. The final output includes two fasta files: coding sequences with and without flanks.

### 2.2 Selecting the best markers and refining the baits design based on gene capture results

**Figure 2:**
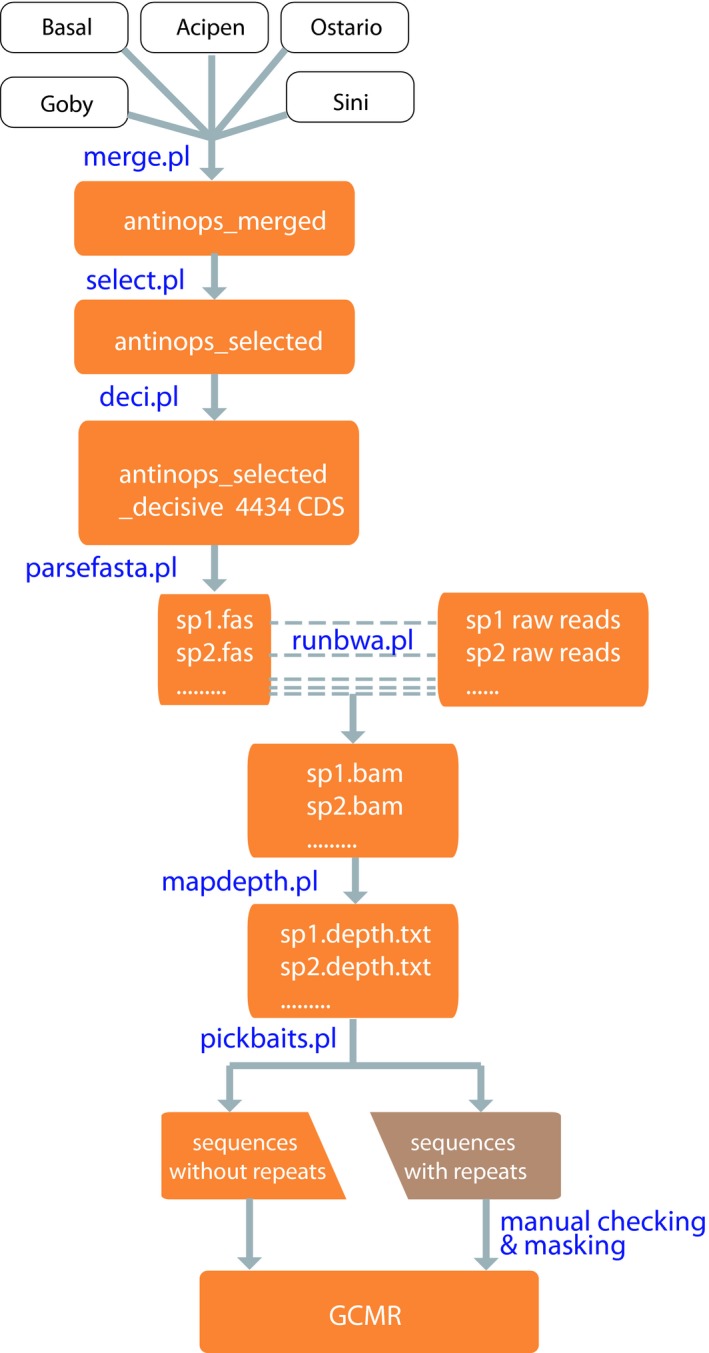
Pipeline of screening for markers with less missing data and better phylogenetic decisiveness and posterior baits refining. I. Merge data from different project (merge.pl); II. select loci with less missing data and high phylogenetic decisiveness (gcmr_select.pl; gcmr_deci.pl); III. find and mask region with extraordinary read depth for bait redesign (parsefasta.pl; runbwa.pl; mapdepth.pl; gcmr_pickbaits.pl). The posterior baits refining steps are optional when empirical data from pilot gene capture are available. GCMR stands for gene capture marker refinement

Based on results of the pilot experiments, exon markers resulting in less missing data were selected, and the baits were evaluated and redesigned with the regions with extraordinarily high read depth were masked (Figure 2). The assembled′ sequences from different projects were merged (*merge.pl*). Briefly, taxa that had more than 3,000 genes captured were kept (*select.pl*). Subsequently, a Perl script *deci.pl* was used to pick phylogenetically decisive loci. Phylogenetic decisiveness means that the datasets should contain all taxa whose relationships are addressed (Dell'Ampio et al., 2014). In our case, the decisive taxonomic groups included eight major clades of the ray‐finned fishes: Acipenseriformes, Lepisosteiformes, Elopomorpha, Osteoglossomorpha, Otomorpha, Gobiaria, Ovalentaria, and Eupercaria. The Polypteridae was excluded in bait design, because both species of the polypterids sampled had fewer than 3,000 targets captured.

From our pilot experiments, we found that partial regions of some target loci had extraordinarily high number of reads mapped, which consumed a large proportion of the total data collected. Those regions escaped RepeatMasker (Smit, Hubley, & Green, 1996) checking in original baits design and wasted a lot of sequencing reads, so we excluded those regions to refine the design of baits. To find those problematic regions, the selected decisive data were parsed to different files by species name (*parsefast.pl*). Then, the raw reads of each species were mapped to the assembled reference sequences of each species using* BWA* (Li & Durbin, 2009). The read depth data were extracted from the mapping results using *SAMtools* (Li & Durbin, 2009) and a custom Perl script (*mapdepth.pl*). Regions with extraordinary high read depth, that is, 100 times greater than adjacent regions were identified and labeled with lowercase letters (*pickbaits.pl*). Loci with these regions were discarded if their length were shorter than 120 bp excluding the masked regions. Longer loci were separated into multiple regions for bait design with the masked regions excluded. To test the utility of refined markers, baits were redesigned based on the result of the pilot experiments and used to enrich and assemble sequences of *Rhinogobius giurinus* for a new round following the aforementioned pipeline.

### 2.3 Testing phylogenetic usefulness of the markers selected and efficacy of the new baits

A phylogeny of 17 species of ray‐finned fishes, including nine species with gene captured data and eight species with sequence data extracted from genomes, was reconstructed. Each individual locus was aligned using Mafft v7 (Katoh & Standley, 2013) with default parameter settings (*mafft_AA.pl*). The aligned AA sequences were translated back to DNA sequences via a custom Perl script *aa2dna_align.pl*. Statistics were summarized from 4,434 aligned loci of nine captured samples and eight species with available genomes. Sequence statistics including average length of coding regions, average GC content, and average pairwise distance (p‐dist) were calculated using a custom Perl script (*statistics.pl*) and R package “ape” (Paradis, Claude, & Strimmer, 2004). Consistency index (CI) and retention index (RI) were calculated using PAUP* v4.0a (Swofford 2003). Due to the high variability of flanking regions, only the coding regions without flanks were used for phylogenetic inference. All aligned loci were concatenated using a custom Perl script (*concatnexus.pl)*. Then, concatenated maximum likelihood (ML) trees were constructed using the ML method implemented in ExaML v3 (Kozlov, Aberer, & Stamatakis, 2015). Concatenated alignments were partitioned by codon and then used to reconstruct the tree under the GTRGAMMA model with 100 bootstrap replicates to assess nodal support.

To test the utility of selected markers for studies at the species level, we reconstructed a species tree of four species of freshwater sleepers (*Odontobutis*, Gobiiformes), whose relationships are unresolved (Ren & Zhang, 2007; Zhong et al., 2017). The species tree was reconstructed based on exon capture data of the chosen markers, including five individuals of each species of *Odontobutis sinensis*, *O. potamophila,* and *O. yaluensis* and one individual of *O. haifengensis*. Two individuals of *Perccottus glenii* were used as the outgroup. ASTRAL v4.11.1 (Mirarab & Warnow, 2015) was used to infer the species tree. An informative unrooted tree cannot be inferred from loci with less than 4 taxa, so these loci were excluded from analyses. Remaining gene trees of each locus were reconstructed using RAxML HPC‐PTHREAD (Stamatakis, 2006) under GTRGAMMA model. Then, they were summarized into species tree using ASTRAL with default parameter settings. Multi‐locus bootstrapping would result in high bootstrap supports even with high discordance among gene trees if there is a sufficient number of genes (Sayyari & Mirarab, 2016), so bootstrap supports were not accessed and branch supports were measured as quartet support instead. Quartet support is the frequency of quartets in gene trees supporting the topology of the species tree and is accessed by implementing option “−t 1” in ASTRAL. A concatenated ML tree was constructed as well. The coding region of each locus was aligned using Mafft v7.294b (Katoh & Standley, 2013) with default parameter setting. Then, aligned loci were concatenated to reconstruct ML trees using RAxML HPC‐PTHREAD (Stamatakis, 2006) under GTRGAMMA model. Nodal support was accessed with 100 bootstrap replicates.

Principal component analysis (PCA) was carried out to visualize inter‐ and intraspecific genetic variation among individuals of the four *Odontobutis* species. As input for the PCA, single nucleotide polymorphisms (SNPs) were extracted from coding regions of the *Odontobutis* data. Loci having data in more than two species were processed with a SNP calling procedure. Reference sequences of filtered loci were generated from aligned sequences based on majority consensus rule using a custom Perl script (*consensus.pl*). Trimmed reads were mapped to the reference using BWA v0.7.15‐r1140 (Li & Durbin, 2009). Picard MarkDuplicates (http://broadinstitute.github.io/picard/) was used to mark duplicates. Then GATK Best Practices of germline short variant discovery recommendations (Van der Auwera et al., 2013; DePristo et al., 2011; McKenna et al., 2010) were followed to do local realignment, base quality score recalibration, SNPs discovery, and genotyping across all samples in concert using standard hard filtering parameters by GATK‐3.2.2 (McKenna et al., 2010). Indels were discarded, and only one of the best SNPs of each locus was selected for downstream analyses to fulfill the assumption of linkage disequilibrium. The vcf file was converted to genotype data file format for PCA by a custom script *vcftosnps.pl*. PCA was performed with R package ade4 (Dray & Dufour, 2007) to unravel variability among 16 *Odontobutis* samples by the *dudi.pca* function.

The new baits refined based on empirical data were compared with the baits designed a priori. The raw reads of each species were mapped to the respective assemblies using* BWA* (Li & Durbin, 2009). The read depth data were extracted from the mapping results using *SAMtools* (Li & Durbin, 2009) and a custom Perl script (*mapdepth.pl*). Reads coverage of each locus was calculated by dividing total length of captured reads by length of the locus. The evenness of read coverage was summarized from a custom designed parameter RC50. Loci were sorted by their read coverage in descending order, and then, the number of loci used half of the total reads is the RC50. Higher RC50 reflects better evenness of read coverage. Read coverage was calculated using a custom Perl script (*coverage.pl*), and RC50 was estimated using *Excel*. The comparison of a priori and a posteriori bait designing was done on capture results for a goby species (*R. giurinus*). Finally, to help researchers to design baits using reference species that are closer to their organism of interest than the eight model fishes that we used, we developed a pipeline of retrieving sequences of the target loci from user‐provided genomes (Supporting information Appendix S3).

### 2.4 Investigate the variability of flanking regions

Since the variability of flanking sequences among different families was too high, we only investigate the variability of flanking regions of the 16 individuals of *Odontobutis*. Sequences with long insertions or deletions, unalignable sequences, and very short flanking sequences (<20 bp) were filtered using a custom Perl script (*flank_filter.pl*, see detailed parameters in Appendix S3). A custom Perl script (*flank_pdis.pl*) was used to summarize length of flanking regions and p‐dist between all pairs of flanking sequences for both filtered and unfiltered flanking regions. SNPs were extracted from filtered coding and flanking regions using GATK following the aforementioned procedure. A number of SNPs in coding and flanking regions were counted with a custom Perl script (*snps_num.pl*). All custom Perl scripts can be found online in Supporting information Appendix S3.

## 3 RESULTS

### 3.1 Single‐copy protein‐coding markers for ray‐finned fishes

The number of loci captured in the pilot experiments ranged from 435 to 11,534 in different samples. All but four samples had more than 3,000 loci captured (Supporting information Appendix S1: Figure S2). The samples that did the worst in gene capture experiment included two polypteriforms (*Erpetoichthys calabaricus* and *Polypterus endlicher*), one sturgeon (*Acipenser ruthenus*), and the Waigeo barramundi (*Psammoperca waigiensis*). After combining the data from all five projects, excluding taxa with fewer than 3,000 loci captured and selecting for phylogenetic decisive loci, we obtained 4,434 CDS markers of 2,261genes. The information of the target loci and sequences of the eight model fish species can be found online in Supporting information Appendix S2.

### 3.2 Phylogenetic usefulness of selected markers

The average length of the coding region of the chosen markers was 236 bp (94–4,718 bp). GC content ranged from 37% to 69% with an average of 55%. Average p‐dist among the 17 species varied from 0.06 to 0.50 substitutions per site, with an overall average of 0.19. Average consistency index (CI) was 0.60 (0.43–0.93), and average retention index (RI) was 0.52 (0.47–0.62) (Supporting information Appendix S1: Figure S3). Maximum likelihood (ML) analyses concatenating 4,434 loci resulted in a well‐resolved tree of major ray‐finned fish clades, and all nodes had 100 bootstrap support values (Figure 1). The resulting phylogenetic tree is consistent with recent studies (Betancur et al., 2013; Faircloth et al., 2013; Hughes et al., 2018), except that the Elopomorpha and the Osteoglossomorpha were found sister to each other and Beloniformes were found more closely related to Blenniformes than to Cichliformes. Because our data only involved a handful of taxa, those inconsistent results should be investigated with better taxon sampling with our exon markers.

There were 4,296 of 4,434 loci captured at least in one *Odontobutis *sample. A total of 1,630 loci were captured in all samples. The average length of target regions was 265 bp (120–5,637 bp). A concatenated ML tree was reconstructed for the four Chinese *Odontobutis* species with *P. glenii* as outgroups, which was well resolved with 100 bootstrap support values for each node. *Odontobutis haifengensis *was sister to the rest of the *Odontobutis* species. *O. yaluensis* was grouped with *O. potamophila,* and *O. sinensis *was placed as sister to them. Individuals of the same species were clustered together. A species tree was also reconstructed with four *Odontobutis* species and *P. glenii*, with a normalized quartet score of 0.64. For the topology of species tree, *O. yaluensis* was also grouped with *O. potamophila*, but the placement of *O. haifengensis *and *O. sinensis* was different (Supporting information Appendix S1: Figure S4). We extracted 36,440 single nucleotide polymorphisms (SNPs) sites from coding regions (35 SNPs per kb) of the 16 *Odontobutis* samples, and only one of the best SNPs from each locus was used for PCA. The PCA showed clear genetic differentiation on interspecific level. Individuals of *O. sinensis *were well separated from other species. Individuals of *O. yaluensis* and *O. potamophila* partially overlapped with each other with respect to PC1 (Supporting information Appendix S1: Figure S5).

### 3.3 Gene capture marker refinement

**Figure 3:**
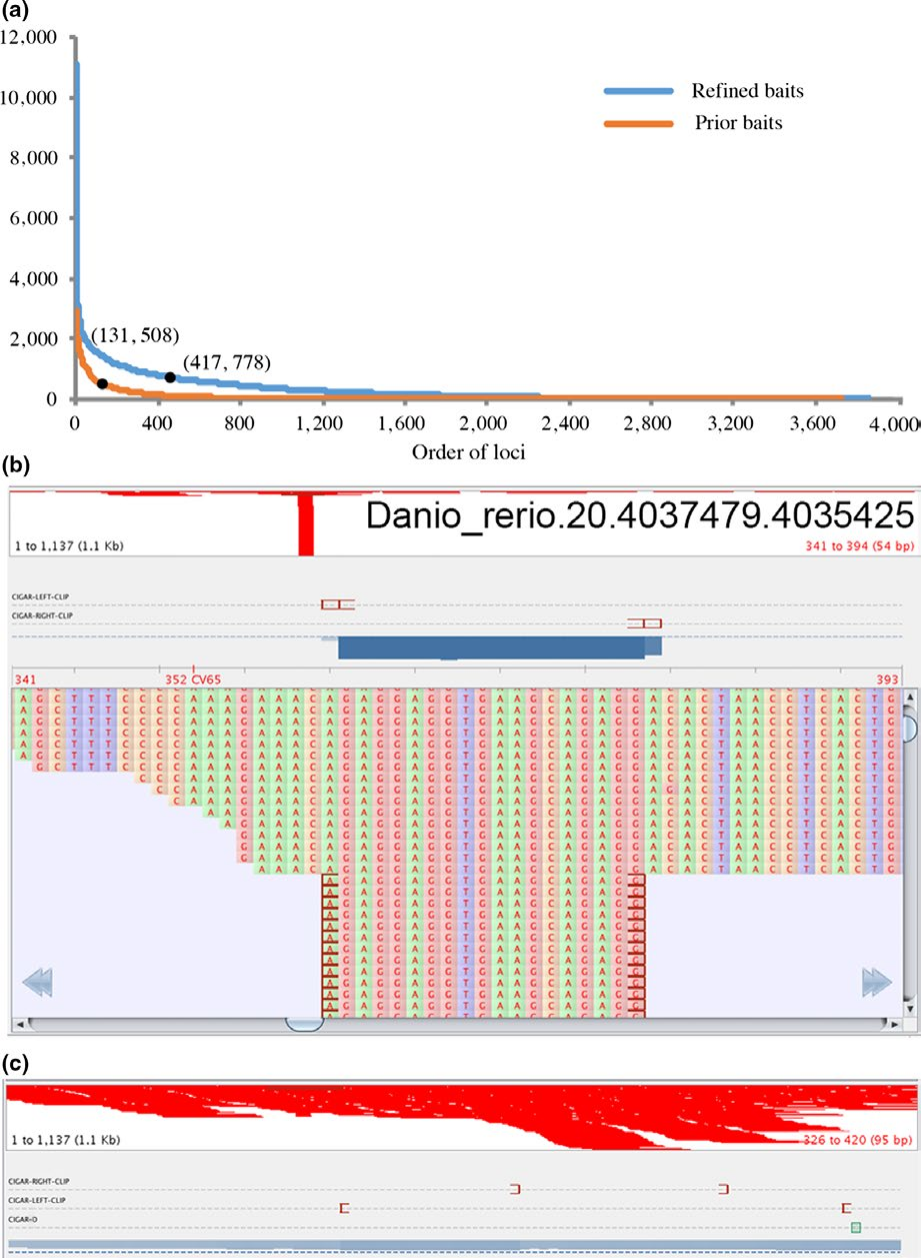
Comparison on evenness of read coverage between results of gene capture using the baits designed a priori (a, blue curve) and the baits refined posteriorly (a, orange curve). (b, c) are screenshots from visualizing the read depth of the locus Danio_rerio.20.4037479.4035425 using Tablet v1.16.09.06. In this example, the result using baits designed a priori (b) is much worse than the result using refined baits (c)

We examined the results of gene capture experiments using original baits. We found that 26 loci of *R. giurinus* had extremely high number of reads mapped. We manually checked those loci and found that all regions with high reads depth had low complexity. We redesigned the baits and carried a new round of gene capture experiment. The gene capture results from new baits had higher RC50 which reflected higher evenness of reads coverage among different loci than the results from the original baits. The reads depths of most of loci using refined baits were higher than the ones using original baits (Figure 3).

### 3.4 Variability of the flanking regions of Odontobutis

**Figure 4:**
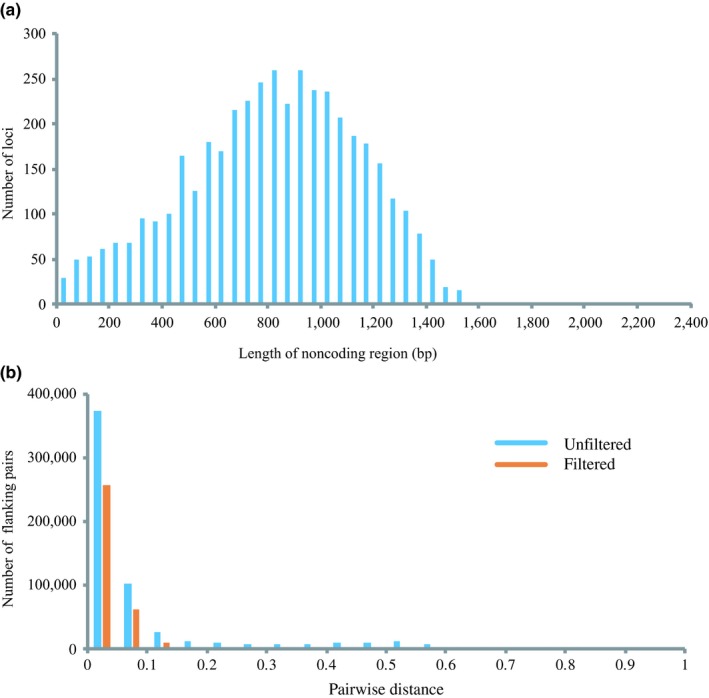
Length distribution of unfiltered flanking sequences of 4,296 loci, from 16 *Odontobutis* individuals (a). Pairwise distance distribution of all pairs of unfiltered (blue bar) and filtered (orange bar) flanking sequences (b)

Length of flanking regions ranged from 0 to 2,271 bp and centered around 800 bp (Figure 4a). P‐dist among them ranged from 0 to 0.84. After filtering unalignable and uninformative short flank regions, p‐dist varied from 0 to 0.57 (Figure 4b). The number of SNPs in the flanking regions was 73,097 (50 per kb), more abundant than in coding regions (36,440 SNPs, 35 per kb).

## 4 DISCUSSIONS

### 4.1 Exon capture

Protein‐coding sequences are more commonly used than noncoding flank regions in phylogenetic analysis. Models of molecular evolution of coding sequences are well studied. Up to 20 popular exon markers, so called “legacy markers” have been used in landmark molecular phylogenetic studies to resolve the tree of life of fishes, long before the target‐capture methods were developed (Betancur et al., 2013; Broughton, Betancur, Li, Arratia, & Orti, 2013; Near et al., 2013, 2012). Our experiments showed that the markers selected and the baits designed were effective in studying phylogenetic relationship of major groups of the ray‐finned fishes, and closely related species as well. The numerous numbers of markers developed here may provide more power to solve the remaining difficult questions in tree of ray‐finned fishes.

### 4.2 A posteriori marker design

The simple repeats in the markers were detected and masked using RepeatMasker by the manufacturer, MYcroarray (Ann Arbor, Michigan) before synthesizing the baits. However, repeats with some variations or complex repeats could not be detected with RepeatMasker, which resulted in a high read depth in some regions (Figure 3b). Extremely high read depth suggested that repetitive regions were enriched to a high degree, which could cause problems in subsequent read assembly, and waste sequencing resources. Based on the sequencing results, we masked these unusual regions in subsequent baits refinement, which produced more even depth for the targeted loci (Figure 3b). If a pilot study is planned before a large‐scale experiment, we recommend applying our method to refine baits design to improve the efficacy of the baits.

### 4.3 Orthology checking and data filtering

Problem of mistakenly using paralogous genes for phylogenetic reconstruction is exacerbated with phylogenomic data, and currently, there is no ideal method to validate orthology of loci assembled from NGS data (Chakrabarty et al., 2017; McCormack, Hird, Zellmer, Carstens, & Brumfield, 2013). The targeted loci we selected for are “single‐copy” (Li et al., 2012), which may have less chance to be paralogous than members of gene families, (Li, Ortí, Zhang, & Lu, 2007). In addition, we performed a “re‐blast” step in data processing pipeline to identify and exclude potential paralogs (Yuan et al., 2016). Nonetheless, both methods cannot guarantee orthology of targeted sequences due to the third round of whole‐genome duplication event in teleosts and slow and steady loss of some paired genes over the subsequent 250 My (Inoue, Sato, Sinclair, Tsukamoto, & Nishida, 2015). Tree‐based methods such as filtering the loci a posteriori based on known monophyly of taxa could be used to alleviate the problem of paralogy.

### 4.4 Phylogenetic utilities of selected markers at species level

The species tree and the concatenated tree reconstructed from 16 *Odontobutis* with two *P. glenii* samples as outgroups showed that the placement of *O. yaluensis* and *O. potamophila* in the two trees was the same, while the position of *O. haifengensis* and *O. sinensis *was conflicting. We found that quartet supports of 3 possible quadri partitions of the clade of *O. yaluensis* and *O. potamophila, O. haifengensis*, *O. sinensis,* and *P. glenii* were 0.39 for topology represented in Supporting information Appendix S1: Figure S5 and 0.31, 0.30 for other two topologies. Close quartet supports for 3 topologies indicated severe incomplete lineage sorting among selected loci, which resulted in the incongruent placement of *O. haifengensis* and *O. sinensis* in species tree and concatenated tree. Nonetheless, concatenated tree still had high bootstrap supports for each node, which indicated high bootstrap value may not reliably reflect accuracy of resulting tree. This finding was also reported in several previous studies (Belfiore, Liu, & Moritz, 2008; Kubatko & Degnan, 2007; Weisrock et al., 2012). For coalescence‐based methods, accuracy can be measured based on concordance between resulting tree and gene trees (Larget, Kotha, Dewey, & Ane, 2010; Sayyari & Mirarab, 2016). So, we recommend a coalescence‐based method to reconstruct species trees and measure accuracy of it with congruence between species trees and the given gene trees. Overall, our results of *Odontobutis* species using the 4,434 loci suggested that those markers can be applied in species‐level applications.

### 4.5 Variability of the flanking regions in Odontobutis

Although we targeted coding regions, flanking sequences were also captured by hitchhiking. Length of flanking sequences was highly correlated with the size of sheared genomic DNA. We could break DNA into longer pieces during library construction if longer flanks were preferred, but inserts >1 kb may sabotage the performance of Illumina sequencing. Some of flanks were nonfunctional sequences and may be less constrained by purifying selection. After filtering unalignable and uninformative short flanking sequences, the amount of remaining data was dramatically decreased, but there were still more SNPs in flanking regions than in coding regions, suggesting that flanking regions may be useful in phylogenetic studies at the species level.

## 5 CONCLUSION

In this work, we developed 4,434 empirically tested protein‐coding markers that are useful in reconstructing phylogenies of the ray‐finned fishes at different taxonomic levels. We also provided researchers with resources for applying those markers in their group of interest: (a) the target sequences of the 4,434 loci for all eight model species which users can use to design baits; (b) a user‐friendly pipeline for users to retrieve target sequences of the 4,434 loci from species of their interest if they provide new genome sequences or transcriptomes; and (c) a pipeline for users to refine their baits design a posteriori based on empirical data. These tools could advance phylogenomic studies in ray‐finned fishes, the most diverse vertebrate group.

## CONFLICT OF INTEREST

None declared.

## AUTHORS CONTRIBUTION

Jiamei Jiang, Hao Yuan, Xin Zheng, Qian Wang, Ting Kuang, Jingyan Li, Junning Liu, Shuli Song, Weicai Wang, Fangyuan Cheng, Hongjie Li, Junman Huang, and Chenhong Li J Jiang analyzed data and wrote the manuscript with support from H Yuan and C Li; X Zheng carried out the experiment and assembled the database with Q Wang, T Kuang, J Li, J Liu, S Song, W Wang, F Cheng, and H Li; J Huang helped in data analyses and wrote scripts for mining target gene sequences from published genomes; and C Li conceived the original idea and supervised all projects.

## Data Availability

Information of 4,434 target loci and respective sequences for all eight model fishes can be found in Appendix S2. The pipeline and scripts for reads assembly, ray‐finned fishes baits design, and refinement are in Appendix S3. The fastq files of raw reads have been deposited in NCBI Sequence Read Archive (SRA) with accession number SRP162615. Accession number of samples referenced from pilot studies can be found in Supporting information Table S2. Sequence alignments in nexus format and input files for analysis were lodged in Dryad with https://doi.org/10.5061/dryad.41j28n0.
