# Supplementary material for: Gene markers for exon capture and phylogenomics in ray‐finned fishes
Source: Ecol Evol. 2019 Mar 5;9(7):3973–83. doi: 10.1002/ece3.5026 (PMC6468074; doi:10.1002/ece3.5026)
Supplement: Supplementary file 3 [file ECE3-9-3973-s003.pdf]

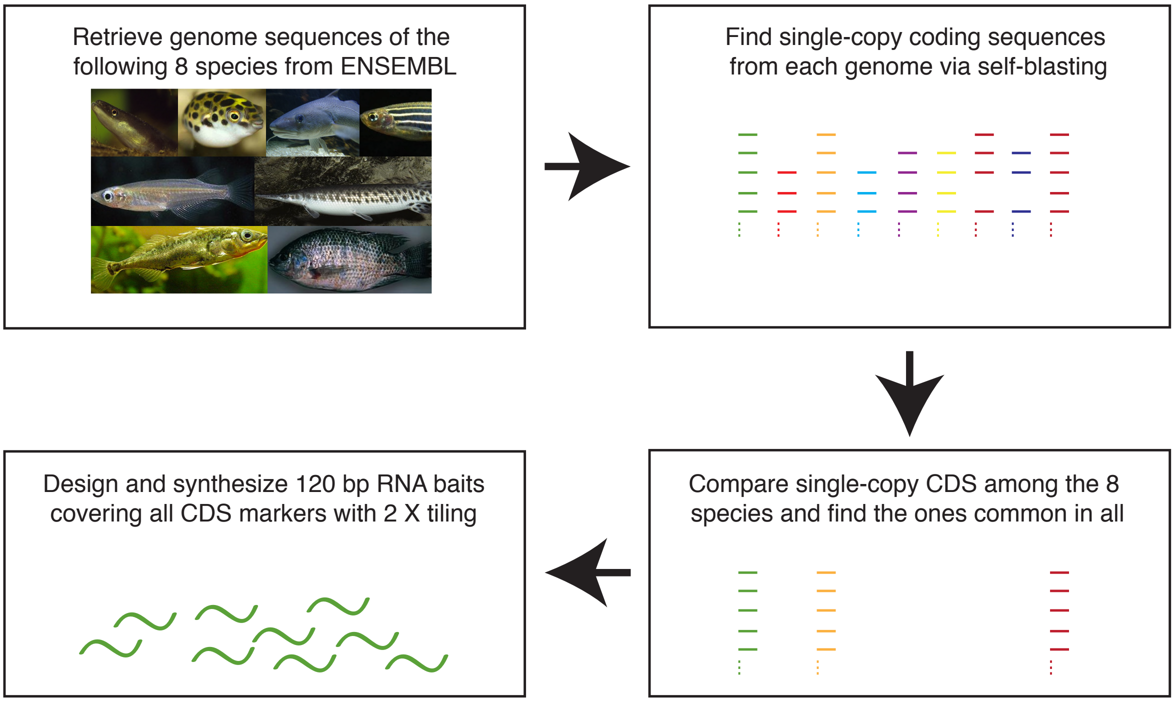

FIG. S1. Mining single-copy protein coding markers through genome comparison using EvolMarkers (Li et al., 2012). The eight species compared are *Anguilla anguilla*, *Tetraodon nigroviridis*, *Gadus morhua*, *Danio rerio*, *Oryzias latipes*, *Lepisosteus oculatus*, *Gasterosteus aculeatus*, *Oreochromis niloticus*.

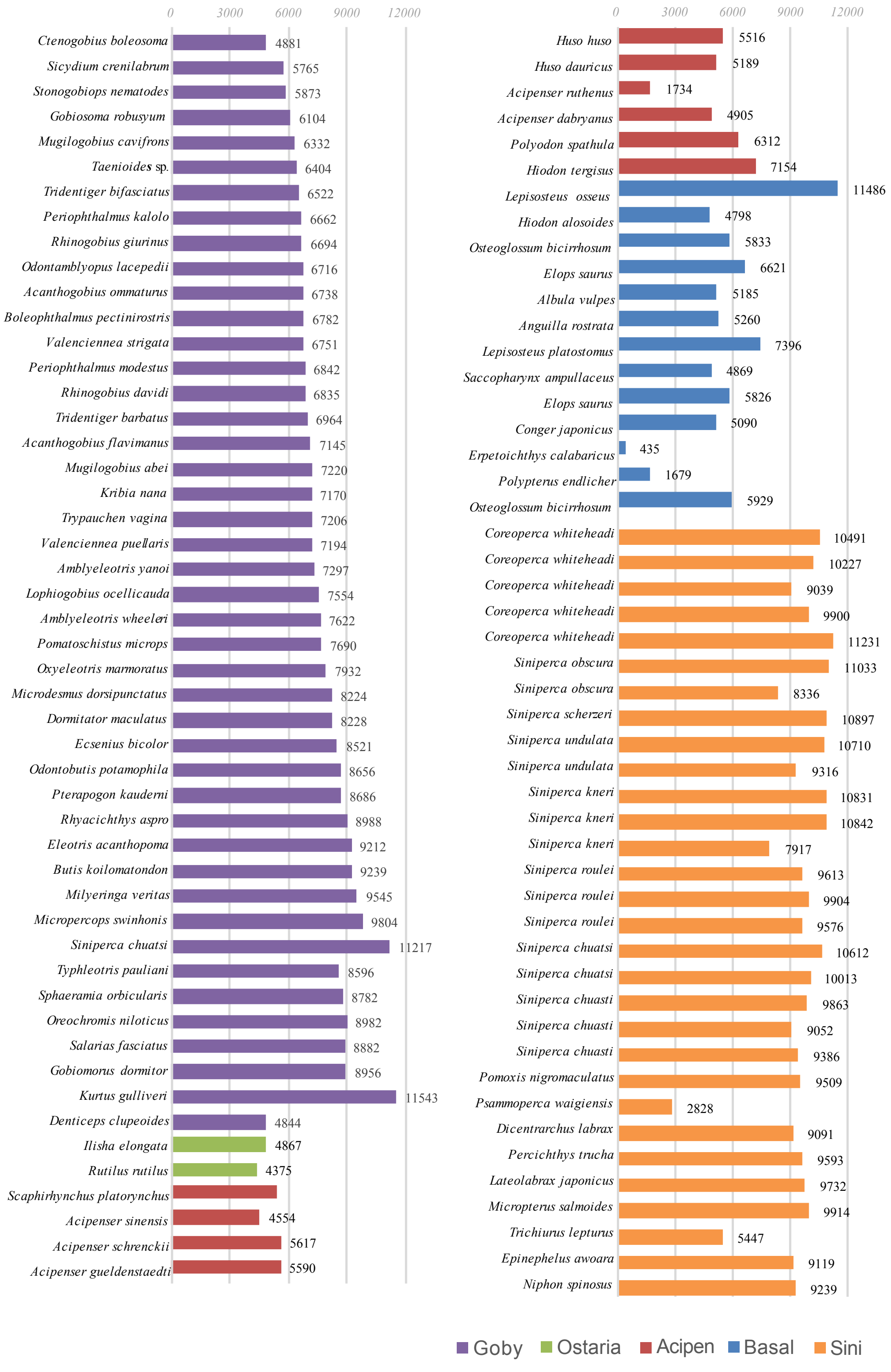

FIG. S2. Number of loci assembled for each sample tested. The color indicates different projects carried in the author's laboratory.

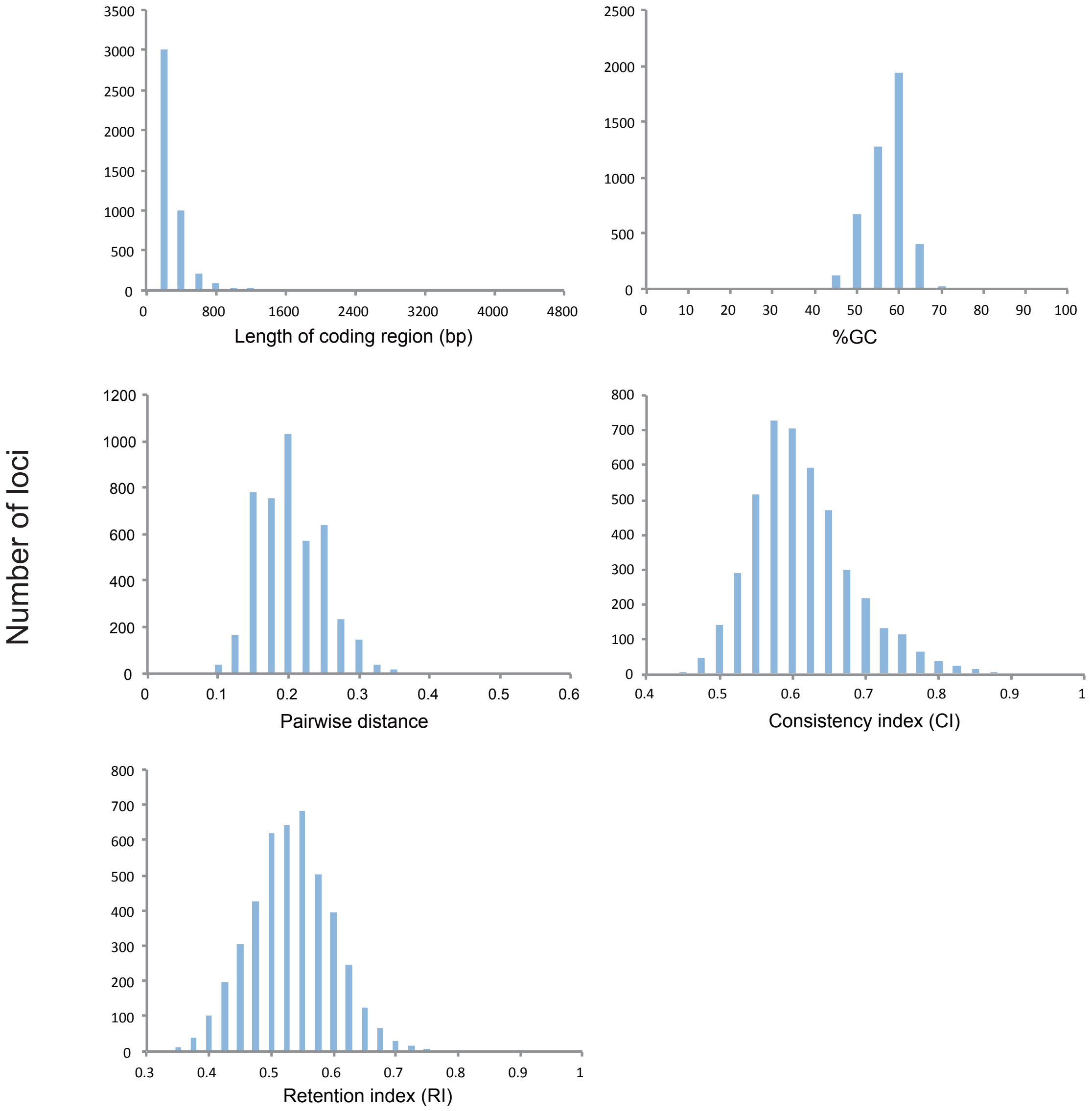

FIG. S3. Average length of coding regions, average GC content, average pairwise distance, retention index and consistency index distribution of 4,434 loci. All statistics were summarized from nine captured samples plus eight species with available genomes.

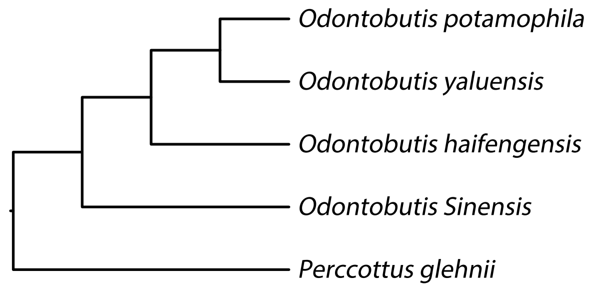

FIG. S4. Species tree of four freshwater sleepers distributed in China reconstructed by ASTRAL v4.11.1 based on 3,817 loci.

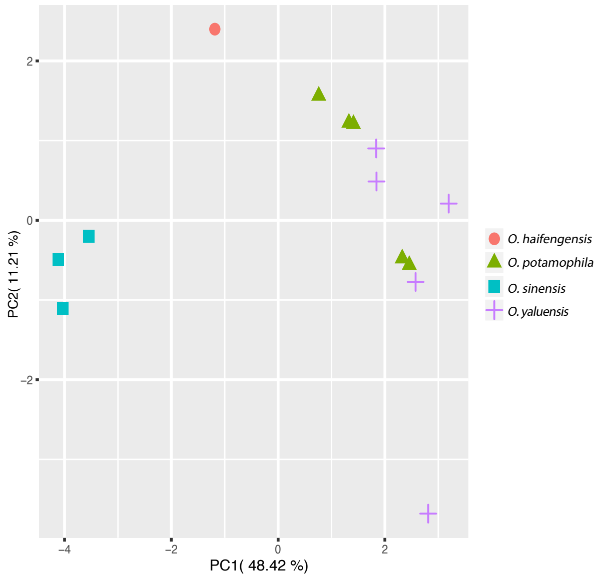

FIG. S5. Principle component analysis (PCA) of four freshwater sleepers (*Odontobutis*) derived from SNPs data of 3,914 loci.
